# Multidisciplinary treatment of esophageal cancer: The role of active surveillance after neoadjuvant chemoradiation

**DOI:** 10.1002/ags3.12350

**Published:** 2020-07-25

**Authors:** Tania Triantafyllou, Bas Wijnhoven

**Affiliations:** ^1^ Department of Surgery Hippocration General Hospital of Athens National and Kapodistrian University of Athens Athens Greece; ^2^ Department of Surgery Erasmus University Medical Center Rotterdam the Netherlands

**Keywords:** active surveillance, esophageal cancer, neoadjuvant chemoradiation, salvage surgery

## Abstract

The optimal treatment of esophageal cancer is still controversial. Neoadjuvant chemoradiotherapy followed by radical esophagectomy is a standard treatment. Morbidity after esophagectomy however is still considerable and has an impact on patients' quality of life. Given a pathologic complete response rate of approximately 30% in patients after neoadjuvant chemoradiation followed by surgery, active surveillance has been introduced as a new alternative approach. Active surveillance involves regular clinical response evaluations in patients after neoadjuvant therapy to detect residual or recurrent disease. As long as there is no suspicion of disease activity, surgery is withheld. Esophagectomy is reserved for patients presenting with an incomplete response or resectable recurrent disease. Active surveillance after neoadjuvant treatment has been previously applied in other types of malignancy with encouraging results. This paper discusses its role in esophageal cancer.

## Graphical abstract

Morbidity after esophagectomy is still considerable and has an impact on patients' quality of life. Given a pathologic complete response rate of approximately 30% in patients after neoadjuvant chemoradiation followed by surgery, active surveillance is currently under investigation as a new alternative approach.

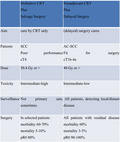

## 1 ESOPHAGEAL CANCER

Esophageal cancer (EC) is an aggressive disease. The two most common types of EC are adenocarcinoma (AC) and squamous cell carcinoma (SCC). AC and SCC differ with regard to etiology, geographic distribution, response to chemotherapy/ radiotherapy, prognosis and possibly need for surgical resection. Esophagectomy is the cornerstone in the treatment of EC. During the last two decades, studies on the lymph node dissection during esophagectomy have shown improved survival in patients who underwent an extensive nodal dissection.^1^ A total number of 23 lymph nodes were proposed as the optimal threshold in order to achieve a maximal survival benefit after esophagectomy. The extent of lymph node dissection expressed as the total number of nodes dissected was found to be an independent predictor of survival.^2^ Whether the observed relationship between the number of nodes dissected and survival reflects a true benefit of more extensive surgery or is due to stage migration, is not clear yet. However, a transthoracic esophagectomy with a two‐field nodal dissection is considered by many as the standard surgical approach nowadays.

Esophagectomy is associated with major complications.^3^, ^4^ The diminished quality of life of patients after neoadjuvant therapy plus esophagectomy is another drawback. A patient’s quality of life is substantially impaired after surgery including role and social functioning.^5^ Reducing morbidity after esophagectomy is a challenge. The application of minimally invasive surgical techniques, better selection of surgical candidates, preoptimization of patient condition, and enhanced recovery protocols have shown to be associated with a reduction in complications and quicker return to normal functioning.^6^, ^7^, ^8^, ^9^, ^10^

## 2 MULTIMODALITY TREATMENT

Perioperative therapies have been incorporated in the treatment of locally advanced EC in the last decade. The rationale is to downstage the disease, facilitate a curative (R0) resection, treat micrometastases and improve overall survival. Studies on SCC mainly originate from Asia whereas AC is mostly seen in the Western world. Besides the published Japanese JCOG9907 and Dutch CROSS studies,^11^, ^12^ an impressive number of (ongoing) randomized controlled trials (RCTs) aim to clarify the benefit and harm of perioperative regimens in the treatment of the disease. There is currently no consensus on the optimal neoadjuvant treatment regimen yet as the CROSS trial (neoadjuvant chemoradiotherapy‐ nCRT), English OEO2 trial (neoadjuvant chemotherapy), MAGIC trial (pre‐ and postoperative chemotherapy), French FFCD trial (nCRT), and German FLOT4 trial (pre‐ and postoperative chemotherapy) all were beneficial but had different regimens.^12^, ^13^, ^14^, ^15^, ^16^, ^17^ The role of neoadjuvant radiation, as an adjunct to chemotherapy, is still questioned by some, especially for esophageal AC. Proponents feel that radiotherapy in the neoadjuvant setting treats both locoregional disease as well as subclinical micrometastases. This is illustrated by the high rate (92%) of patients that underwent radical surgery (resection margins negative) after nCRT.^12^ Moreover, almost one third of the patients after nCRT in the CROSS trial had a pathologically complete response (pCR), i.e. no viable tumor cells in the resection specimen. This opens the way to think about the concept of an organ sparing treatment for EC.

## 3 THE CONCEPT OF ORGAN‐PRESERVATION

Thorough understanding of the impact of neoadjuvant therapies on rectal cancer patients led to the hypothesis that radiation may be responsible for increasing tumor necrosis over time justifying a less extensive resection.^18^ Prolongation of the time interval between nCRT and surgical resection supported the rationale for preservation of the anal sphincter. Although the initial goal of nCRT was to facilitate a radical surgical resection and decrease rates of locoregional recurrence after surgery, the observation of a clinically complete response (i.e. no proof of residual tumor by clinical staging modalities including endoscopy and imaging techniques; cCR) after neoadjuvant therapy in a proportion of cancer patients who were unfit for surgery led to an active surveillance or “wait and see” policy. Herein, systemic and local treatment can lead to regression of the primary tumor, while control of undetectable micrometastases at time of diagnosis might also be another benefit. Resection of the primary tumor and locoregional lymph nodes is reserved for patients with residual/recurrent disease only. Standard surgery is now omitted from the multimodal treatment in several types of malignancy, as CRT alone was found to be curative in some patients with bladder, prostate, head and neck, and rectal cancer.^19^, ^20^, ^21^, ^22^, ^23^, ^24^, ^25^

## 4 ORGAN‐PRESERVATION IN ESOPHAGEAL CANCER

The CROSS study showed improved overall and disease‐free survival after five weekly cycles of carboplatin and paclitaxel with concurrent 41.4 Gy radiation plus surgery for patients diagnosed with locally advanced EC.^12^, ^13^, ^26^ Distant recurrence rates were also lower for patients that underwent combined treatment compared to patients that underwent esophagectomy alone. Furthermore, the CROSS study showed that nearly one third of the patients had a pCR: 49% in SCC and 23% in AC.^12^, ^26^ This finding fueled the debate on applying active surveillance after nCRT. Theoretically, patients with a cCR (based on endoscopy with biopsies, endosonography (EUS), positron emission (PET) and computer tomography (CT) scanning) may have been cured (i.e. have a true pCR) and could potentially be spared an esophagectomy. A second possible benefit of an active surveillance strategy in patients with a cCR is that tumors with an aggressive biological behavior and yet undetected disseminated disease that cannot be cured with surgery will be identified over time before recurrent local disease becomes detectable. The main argument supporting an organ‐sparing approach in this group of patients is that, despite surgery, early systemic recurrence will occur (within 1 year) and surgery for local disease control is not needed; therefore, patients are put at risk for morbidity and mortality of an operation without changing prognosis.^27^, ^28^ In other words, avoiding unnecessary major surgery at a time when distant metastases are present but cannot be detected may result in similar oncologic outcomes with a high likelihood of improved quality of life and preservation of immune system activity.

The feasibility of an active surveillance approach for EC has been investigated in a step‐by‐step process. Shapiro et al concluded that a prolonged time to surgery up to 45 days after nCRT had no effect on disease‐free and overall survival.^29^ Interestingly, postponed surgery up to 12 weeks not only did not affect the oncologic outcome but increased the probability of a pCR. The importance of delaying surgery up to 12 weeks after nCRT is that this allows for a more accurate assessment of a cCR by endoscopy, EUS, and PET‐CT scanning. By 12 weeks post‐surgery, most inflammatory changes due to CRT have largely resolved. Another retrospective study found no differences in postoperative complications or survival between patients operated on less or more than 8 weeks after neoadjuvant treatment.^30^ Some other studies found that a time interval of at least 10 weeks for AC and 13 weeks for SCC after completion of neoadjuvant treatment was associated with a higher probability of pathologic pCR.^31^, ^32^

As it was felt that a response assessment would be optimal 12 weeks after nCRT, the next question was what modalities are best for clinical response assessment? And if there is residual disease in the esophagus, where is this located and can this be targeted? To answer these questions, the resection specimens of 102 consecutive patients after nCRT and esophagectomy were evaluated. In non‐complete responders (i.e. residual cancer in the resection specimen after nCRT), 89% of the patients had residual tumor cells in the mucosa and/ or the submucosa.^33^ Hence, concentric and toward the lumen regression seem to compose a mixed pattern of residual disease despite lack of involvement of the surrounding stroma and regional lymph nodes. This finding may allow a safe and reliable follow‐up based on both endoscopic (with biopsies) and imaging modalities. The accuracy of diagnostic tests for the assessment of a cCR has been evaluated in the preSANO (Surgery As Needed for Oesophageal Cancer) trial, which was designed as a prospective, single arm, multicenter trial.^34^ The clinical response evaluation (CRE) was proposed as a two‐ step process (CRE I and II) in six centers in the Netherlands. Patients diagnosed with either AC or SCC receiving the CROSS regimen underwent the first evaluation (CRE I) consisting of endoscopy with bite‐on‐bite biopsies and EUS 4‐6 weeks after completion of nCRT. The second evaluation (PET‐CT, endoscopy with biopsies, EUS and fine needle aspiration (FNA) of suspicious lymph nodes; CRE II) included patients with no findings of residual disease during CRE I. All patients eventually underwent surgery. The primary endpoint of this study was the association of cCR with pCR (pathological assessment of the resection specimen).

In this study, 31% of tumor regression grade (TRG) 3 or TRG4 (>10% residual carcinoma in the resection specimen) were missed by endoscopy with regular biopsies and FNA, 10% were missed by bite‐on‐bite biopsies plus FNA, 28% were missed by EUS plus FNA, 15% were missed by PET‐CT.^35^ Sensitivity of endoscopy alone hardly exceeds 60% in the existing studies.^36^, ^37^ Cheedella et al compared cCR to pCR in one of the largest cohort studies published.^38^ Two hundred and eighty‐four patients with EC were evaluated after nCRT. Among the 77% of patients with cCR after nCRT, only 31% achieved pCR after surgery. Overall, sensitivity of cCR for pCR was 97.1%, but specificity was too low (29.8%). These findings confirm that preoperative staging remains one of the biggest challenges in the management of EC albeit the evolving technologic advances. Focusing on the role of endoscopic biopsies, the preSANO study proved that bite‐on‐bite biopsies increased the chance of detecting residual cancer cells in deeper layers of the esophagus, such as the submucosa, compared with regular biopsies.^34^ Moreover, at least two independent expert pathologists revised each endoscopic and surgical specimen, while the learning curve of accurate endoscopy and precise pathologic examination seems to improve over time based on strict protocols and technologic novelties.

A side study of the preSANO trial revealed the inaccuracy of the PET‐ CT for the identification of TRG3‐4 and the inability to distinguish relapse of the disease from inflammation at 12 weeks post‐ nCRT.^39^ However, distant metastases were detected in almost 10% of patients and surgery was withheld in this group. These patients would otherwise be operated on when no PET‐CT was performed 12 weeks after nCRT. Hence, PET‐CT is useful for the detection of interval metastases and may have a role in an active surveillance strategy with serial scanning. According to a recent meta‐analysis, endoscopic biopsies, EUS, PET‐CT, and PET‐CT with SUVmax or %DSUVmax identified residual disease with a sensitivity of 33%, 96%, 74%, 69%, and 73% and specificity of 95%, 8%, 52%, 72%, and 63%, respectively.^40^ Although EUS presents the highest sensitivity among the other tests and endoscopic biopsies are followed by a significant specificity, the use of all tests increases the possibility of early detection of residual or regrowth disease during the follow‐ up period.

## 5 DEFINITIVE CHEMORADIATION PLUS SALVAGE SURGERY VERSUS NEOADJUVANT CHEMORADIATION AND SURGERY AS NEEDED

The idea of CRT without surgery, also called definitive CRT (dCRT), for EC is not novel. Observational studies including patients with unresectable tumors or patients not eligible for a surgical resection due to limited physical status underwent dCRT with the aim to achieve cure without surgery. A French RCT showed that patients diagnosed with locally advanced cancer of the thoracic esophagus, mainly SCC, who respond to CRT do not benefit from additional surgery compared to continuation of CRT therapies (definite treatment).^41^ This was also shown in a Chinese RCT.^42^ The 5‐year overall survival was comparable between the group that underwent surgery vs patients with dCRT. A third RCT compared the efficacy of induction chemotherapy plus CRT (40 Gy) plus surgery to induction chemotherapy plus dCRT (at least 60 Gy) without surgery. This study concluded that despite improved local control, surgical resection did not affect survival in patients with locally advanced SCC.^43^ Nowadays, in patients with SCC, dCRT is considered a curative treatment, especially in patients that are not good surgical candidates.

A phase‐II study evaluated the results of dCRT for resectable locally advanced EC in 41 patients. Some 28 (68%) patients had grade 3 or higher toxicity, while four therapy‐related deaths were recorded reflecting the toxicity of the regimen. Twenty‐one patients underwent surgery for residual or recurrent disease where dCRT had not cured the disease.^44^ Additional esophagectomy after dCRT in patients with residual/recurrent cancer is defined as *salvage* surgery. Surgery after dCRT should be considered as a “rescue” treatment rather than *delayed* surgery as proposed in the active surveillance protocols after neoadjuvant therapy.

A retrospective multicenter European study compared patients who underwent salvage esophagectomy after dCRT with patients who underwent planned esophagectomy after completion of nCRT. Interestingly, anastomotic leak and surgical site infection rates were higher after salvage surgery while 3‐year overall and disease‐free survival were similar for the two groups.^45^ nCRT (up to 40‐45Gy radiation) is associated with lower complication rates and less toxicity as compared to dCRT (50‐60Gy). Secondly, surgery is likely associated with less complications given the lower dose of radiation applied resulting is less mediastinal fibrosis. Limiting the dose and field of radiation may also limit cardiac and pulmonary toxicity reducing postoperative surgical and medical complications. Finally, there is no strong evidence from randomized clinical studies that nCRT regimes are less effective than the dose of radiation used for dCRT in terms of pathological response and survival. Therefore, an organ‐ sparing approach using a nCRT regimen with surgery as needed in patients that have residual or recurrent disease seems reasonable. Whereas in dCRT the aim of the treatment is to cure the disease by not applying surgery, in a “surgery as needed” approach resection is still anticipated but may not be needed in patients with a persistent cCR. Table 1 shows the differences between the two treatments.

**Table 1:** Differences between definitive chemoradiation with salvage surgery and neoadjuvant chemoradiation with delayed surgery (surgery as needed)

	Definitive CRT Plus Salvage Surgery	Neoadjuvant CRT Plus Delayed Surgery
Aim	cure by CRT only	(delayed) surgery cures
Patients	SCC Poor performance cT4	AC‐SCC Fit for surgery cT1b‐4a
Dose	50.4 Gy or>	40 Gy or>
Toxicity	Intermediate‐high	Intermediate‐low
Surveillance	Not primary aim, sometimes	All patients, detecting local/distant disease
Surgery	In selected patients morbidity 60%‐70% mortality 5%‐10% pR0 80%	All patients with residual disease morbidity 60% mortality 3%‐5% pR0 90%‐100%

## 6 RETROSPECTIVE STUDIES ON THE EFFICACY OF ACTIVE SURVEILLANCE IN ESOPHAGEAL CANCER

A Dutch multicenter study of 31 patients under active surveillance with surgery as needed and 67 patients in the immediate surgery group after nCRT (CROSS regimen) showed that 3‐year overall survival was 77% and 55%, respectively (HR 0.41; 95% CI 0.14‐1.20, *P* = .104).^46^ Moreover, the 3‐year progression‐free survival was 60% and 54%, respectively (HR 1.08; 95% CI 0.44‐2.67, *P* = .871). Importantly, distant dissemination rate, R0 resections, and postoperative complications were comparable between the two groups. However, this was a retrospective study in which the median follow‐up of the active surveillance group was less than 3 years. Another drawback was the heterogeneity in the surveillance strategies.

The MD Anderson Cancer Center presented their experience with surgery in patients with a cCR who underwent surveillance. The 5‐year overall survival was 58%. Twelve of 13 patients that had a locoregional regrowth could be operated on (delayed surgery) with excellent perioperative outcomes. Comparison of these patients with patients undergoing standard treatment (neoadjuvant therapy plus surgery irrespective of response to treatment) showed no statistically significant difference in median overall survival.^47^, ^48^ Similar studies comparing survival after active surveillance plus delayed surgery in patients with a cCR and standard treatment (neoadjuvant CRT plus standard surgery) come from Ireland and Italy, and support an active surveillance strategy.^49^, ^50^

On the contrary, a French retrospective study found a higher recurrence rate when surgery was omitted after CRT (50.8% vs 32.7%, *P* = .021).^51^ In this study, the vast majority of the patients had a SCC (84.1%). Patients who underwent additional esophagectomy also had a higher 5‐year overall survival compared to the non‐operative group. Although it appears that these results were in favor of the operative approach over surveillance, selection bias of the patients who were included in the study and underwent surgery is a major limitation of the study. For instance, patients after dCRT who refused to undergo surgery and were included in the surveillance group could have a poor physical status. Indeed, patients that underwent surveillance were older, had higher age, more often a poorer nutrition status, and higher ASA score. Moreover, neoadjuvant setting and dosages were heterogeneous.

In summary, these studies support the feasibility and safety of an active surveillance approach in selected patients with a cCR after nCRT, and this is in line with a recent systematic review from the Netherlands.^52^ The decision of a nonoperative strategy is also supported by patients’ preference according to a recent study. It was shown that patients accept a lower chance of overall survival in order to avoid an esophagectomy.^53^

## 7 RANDOMIZED CLINICAL STUDIES

The Dutch SANO trial is a phase III multicenter RCT aiming to compare the clinical and oncologic outcome of neoadjuvant therapy with surgery as needed/active surveillance versus neoadjuvant therapy plus standard esophagectomy in patients with resectable AC or SCC.^54^ The trial seeks to prove non‐inferiority of active surveillance compared to standard surgery. The primary outcome of the study is overall survival. Secondary outcomes are the proportion of patients who do not undergo surgery, quality of life, irresectability (T4b) rate, radical resection rate, postoperative complications, progression‐free survival, distant dissemination rate, and cost‐effectiveness. In the intervention arm (active surveillance), patients with a cCR 12 weeks after nCRT will undergo intense follow‐up (CREs) and (delayed) surgery is only done when there is a strong suspicion of cancer recurrence without distant metastases. In further detail, during CRE‐I 6 weeks after completion of induction CRT (CROSS), all patients undergo esophagoduodenoscopy with biopsies, radial EUS with additional EUS‐FNA in case of suspected lymph node disease, and PET‐CT for exclusion of distant metastases.^46^ CRE‐II is performed 12 weeks after completion of CRT including 18F–FDG‐PET‐CT, followed by endoscopy with bite‐on‐bite biopsies and ultra‐endosonography plus fine needle aspiration of suspected lymph nodes and/or PET‐positive lesions. Complete responders are then encountered in the active surveillance therapeutic arm and are scheduled to undergo intense CREs (PET‐CT, bite‐on‐bite biopsies, EUS‐FNA) every 3 months during the first year, every 4 months during the second year, twice a year for the third year and annually for the fourth and fifth years. The randomization process is rather innovative as it follows a random sequential switch of clusters of participating centers among the two therapeutic arms every 4.5 months; a so‐called stepped‐wedge cluster design. Recruitment is ongoing and planned to be completed mid‐2020. “Another prospective multicenter diagnostic cohort study applies the same protocol performing CREs in patients diagnosed with SCC. Recruitment is currently ongoing in four Asian centers.”^55^

The French Esostrate‐Prodige 32 study is also comparing standard surgery with active surveillance after nCRT for resectable EC.^56^ Randomization, in contrast to the SANO trial, is done on an individual and not institutional level. Moreover, the Esostrate trial uses a more intense neoadjuvant treatment. Therefore, the pCR may be higher than in the SANO trial, albeit the possibility of higher risk of toxicity and increased adverse effects. The primary outcome is overall survival. Recruitment is slow, however.

The design of the SANO trial seems to facilitate a smooth recruitment of patients among the 12 participating high‐volume centers. Randomization on an individual rather than an institutional level has some limitations as pointed out by Blazeby et al. They concluded that optimizing recruitment of patients towards an operative vs a nonoperative approach appears to be challenging. Only 11% of the patients with SCC were finally eligible for randomization in a feasibility study on dCRT vs surgery.^57^ This was attributed to the discrepancy during the informative process for consent of the patients between the centers performed by surgeons and oncologists. Audio‐recording consultations, data interpretation, outcome analysis, and training of recruiters may be the key in further enhancing randomization in demanding oncologic hypotheses to be investigated.

## 8 POTENTIAL BENEFIT OF ACTIVE SURVEILLANCE

A recently published international study on 2704 patients diagnosed with EC who underwent esophagectomy between the years 2015‐2016 disclosed a 59% overall incidence of complications.^58^ Moreover, 30‐ and 90‐day mortality was 2.4% and 4.5%, respectively. Interestingly, the vast majority of patients with a complication experienced multiple adverse events. The comprehensive complication index (CCI) was developed in an effort to summarize the total burden of postoperative complications in a single comprehensive parameter. In a later analysis of the CROSS trial, the CCI was comparable for patients who underwent nCRT plus surgery vs surgery alone.^59^ However, patients after esophagectomy experience long‐lasting symptoms impacting on quality of life.^60^, ^61^ Alimentary disorders and reflux are the most frequent symptoms reported.^61^ Overall, nutritional and psychological status are strongly deteriorated after surgery mainly due to changes of daily habits, while fatigue and appetite loss may persist for a long period postoperatively.^60^ This justifies initiating studies looking at the benefit and harm of "surgery as needed" since avoiding an esophagectomy will not put the patient at risk for a reduced quality of life. Secondly, there is no risk of morbidity and mortality related to the surgical intervention as previously reported in other malignancies.^20^, ^62^, ^63^

Another argument towards delaying surgery after nCRT and opting for an organ‐sparing approach is that patients have more time to recover after therapies with improvement of physical, social, and self‐care. Surgical trauma and its consequences also impair the immune system.^63^, ^64^, ^65^, ^66^ Hence, avoidance of surgery may provide time for the immune function to self‐ reinforce and attack any possibly remaining viable tumor cells. Finally, as already discussed, prolonged time to surgery was associated with a better histopathological assessment of tumor response to neoadjuvant treatment and prognostication.^29^

## 9 CONCERNS

The accuracy of diagnostic tests used during active surveillance is a possible concern. Residual cancer after nCRT may be missed and this may lead to an unnecessary delay of surgery in patients with false negative CREs. Theoretically, this could lead to patients presenting with an irresectable or incurable (cT4b) regrowth or a lower chance for a complete tumor resection (R0). It also remains unknown if delayed surgery increases postoperative morbidity or mortality and distant dissemination rate. However, the lower dose of radiation, close and repeated monitoring of patients for disease recurrence and patient selection are more favorable in an active surveillance approach than salvage surgery after dCRT. One may also argue that there is a chance for a higher distant dissemination rate in patients that undergo active surveillance. Undetected residual cancer cells may give rise to blood‐borne metastases. These concerns have been addressed in the protocol of the SANO‐study and appropriate stopping rules have been defined.^54^

The expertise of physicians involved in the response evaluations and interpretation of data is important and implementing an active surveillance program needs to be guided, guarded, and supported by a health system. Dedicated multi‐disciplinary team meetings, repeated quality assessments, and training of the staff involved is important. Although the active surveillance approach in EC may result in non‐inferior overall survival and lower treatment‐related morbidity, the cost‐effectiveness of this treatment approach is yet unknown. In summary, close monitoring is needed for patients in an active surveillance program. This involves the repeated use of accurate diagnostic modalities and skilled and trained specialists in order to prevent irresectable or incurable regrowth of cancer that may even give rise to distant metastases.

## 10 FUTURE PERSPECTIVE

The value of the additional role of diffusion‐weighted (DW) technology in T2‐weighted (T2W) MRI has been recently presented. Sensitivity and specificity of this combined technique increased from 90%‐100% and 8%‐25% to 90%‐97% and 42/50%, respectively ^67^. Despite the low specificity and the risk of over staging complete responders, it is undoubtedly a trustworthy tool that can be incorporated in the current protocols of active surveillance in order to improve early detection of residual or recurrent disease. Another novel tool that may be implemented in the surveillance protocols in the future is the circulating tumor DNA (ct‐ DNA) technology. This, along with new biomarkers identified in the peripheral blood, may have the potential to contribute to the earlier and more accurate detection of dissemination of the disease‐identifying targeted genetic markers.^68^, ^69^

## CONFLICT OF INTEREST

Authors declare no conflict of interests for this article.
